# Efficacy of topical bevacizumab 0.05% eye drops in dry eye disease: A double-masked, randomized trial

**DOI:** 10.1371/journal.pone.0234186

**Published:** 2020-06-05

**Authors:** Ngamjit Kasetsuwan, Kanawat Chantaralawan, Usanee Reinprayoon, Lita Uthaithammarat

**Affiliations:** 1 Department of Ophthalmology, Faculty of Medicine, Chulalongkorn University and King Chulalongkorn Memorial Hospital, Bangkok, Thailand; 2 Center of Excellence for Cornea and Stem Cell transplantation, Department of Ophthalmology, Faculty of Medicine, Chulalongkorn University and Excellence Center for Cornea and Limbal Stem Cell Transplantation, Department of Ophthalmology, King Chulalongkorn Memorial Hospital, Thai Red Cross Society, Bangkok, Thailand; 3 Department of Anatomy, Faculty of Medicine, Chulalongkorn University, Bangkok, Thailand; Xiamen University, CHINA

## Abstract

The objective of this double-masked, placebo-controlled, randomized trial was to assess the efficacy and safety of bevacizumab 0.05% eye drops in dry eye patients. This study included Dry Eye Workshop Study (DEWS) Grade 3–4 dry eye participants (n = 31) whose tear break-up time (TBUT) was ≤5 seconds(s). Participants were randomized to undergo treatment with either bevacizumab 0.05% eye drops (n = 19) or placebo (n = 12). The primary outcome was TBUT, and the proportion of responders (increase of ≥3s in TBUT at week 12), ocular surface disease index (OSDI) score, Schirmer test, and Oxford scheme grade were secondary outcomes. All outcomes were measured at 1-, 4- and 12 weeks. TBUT in bevacizumab group differed significantly from TBUT in placebo group within 12 weeks (P = 0.001). Moreover, the improvement of TBUT in bevacizumab group versus placebo group at 4- and 12 weeks differed significantly from that difference at baseline (P = 0.002 and P = 0.003, respectively). The proportion of participants achieving increase of 3 seconds or more of TBUT at week 12 in the bevacizumab group was significantly greater than that in the placebo group (P = 0.02). Oxford scheme grade at 1-, 4- and 12 weeks differed significantly from the values at baseline in bevacizumab group (P = 0.001, P = 0.01, and P = 0.03, respectively). OSDI scores at 1-, 4- and 12-week follow-ups were significantly lower than that at baseline in bevacizumab group (P<0.001 at each follow-up). Schirmer test were not significantly different within or between groups (the lowest P = 0.92). No adverse events occurred in this study. Patients treated with bevacizumab 0.05% eye drops showed significant improvement in tear film stability, corneal staining and symptoms.

## Introduction

Dry eye disease (DED) is a chronic inflammatory ocular surface disease resulting in various symptoms, including ocular surface irritation or pain, eye redness and epiphora. According to the Tear Film and Ocular Surface: Dry Eye Workshop Study II^™^ (TFOS DEWS II^™^), the definitive treatment for DED is still not known, due to DED’s unknown pathogenesis. Evidence of inflammation in DED has been shown as increased secretion of inflammatory cytokines, such as interleukin (IL)-1*α*, IL-1β, tumor necrosis factor *α* (TNF*α*) and vascular endothelial growth factor (VEGF), which leads to tear hyperosmolarity. Inflammation also causes neovascularization, which, in turn, produces more pro-inflammatory cytokines, making a vicious cycle [1–4]. Therefore, the definitive treatment for DED would be to remove one of the factors in this cycle.

Jiang et al. found that patients with DED who underwent subconjunctival bevacizumab (anti-VEGF-A) injection had a better tear break-up time (TBUT) and fewer symptoms than did patients in a control group [2]. VEGF, acting as a pro-inflammatory cytokine, plays an important role in neovascularization and could promote other pro-inflammatory cytokines, such as TNF*α* and IL-6, in the process of inflammation [5]. Moreover, Cursiefen et al. stated that VEGF-A is an essential hemangiogenic and lymphangiogenic factor [6]. This is hypothesized to result from the recruitment of macrophages, which can further secrete VEGF-C/-D to amplify the hemangiogenesis and lymphangiogenesis processes involved with immunopathogenesis and the vicious cycle of DED [6, 7]. In 2009, Koenig et al. found that bevacizumab eye drops could effectively inhibit neovascularization in both cultured corneal cells and *in vivo*, in a pilot study [8]. Despite the development and increasing trend in usage of bevacizumab eye drops, trials powered to assess the efficacy and safety of topical bevacizumab eye drops in DED are still lacking.

We aim to study the effectiveness of bevacizumab 0.05% eye drops in DED as a possible novel treatment.

## Materials and methods

This was a prospective, randomized, doubled-masked placebo-controlled clinical trial (Thai Clinical Trials Registry, TCTR 20171024002) at the Department of Ophthalmology, Faculty of Medicine, Chulalongkorn University. The study was approved by the Faculty of Medicine, Chulalongkorn University’s institutional review board (IRB no. 074/60) at 18 May 2017, and adhered to the tenets of the Declaration of Helsinki. The authors confirmed that all ongoing and related trials for this drug/intervention were registered. This study was carried out from 17 June 2017 to 19 November 2017 which was started 1 month after approval from the institutional review board but before the approval from TCTR at 19 October 2017 because we were preparing and contacting for registering this trial right after approval of the institutional board review. However, this study was the project for completing residency training of K.C. Since we did not know the exact time of the process of TCTR would take, we afraid that it would not be in time for submitting this project for passing residency program if we started after the approval of TCTR.

### Participants

Participants were recruited from the outpatient clinic of the Department of Ophthalmology, King Chulalongkorn Memorial Hospital, and evaluated for the eligibility criteria. Inclusion criteria comprised the following: age between 18 and 80 years; TBUT ≤ 5 seconds; ability and willingness to comply with the treatment/follow-up schedule and requirements; and ability to provide informed consent. Exclusion criteria included: presenting with mild or moderate DED condition (severity level 1 or 2 according to DEWS 2007 report [1]); having history or presence of non-DED ocular surface disorder or structural abnormalities involving tear secretion or evaporation i.e. trichiasis or entropion; having any other current active eye disease other than DED that required the use of ophthalmic medication; presence with pterygium or pinguecula; any inflammation in the iris or anterior chamber; glaucoma; systemic conditions that affect the health of the ocular surface; history of bevacizumab contraindication; using any topical medication other than artificial tears within the past 3 months; using drugs that may interfere with tears production, i.e. anti-depressive, anticholinergics, anti-histamine medication, antihypertensive, calcium channel blocker, antacids, systemic corticosteroids, and retinoids within the past 3 months; and previous ocular surgery or contact lens use within the past 6 months. Female of child bearing potential were excluded from participation in the study if they were pregnant.

### Study protocol

The investigational eye drops, supplied as a sterile, preservative-free, clear aqueous solution containing 0.05% bevacizumab, were prepared from standard bevacizumab solution (Avastin, Genentech Inc, South San Francisco, CA) for intravenous infusion, diluted in 0.9% normal saline solution at the hospital’s Pharmacy Department. Placebo was prepared from 0.9% normal saline solution alone.

All participants who met the eligibility criteria were asked to give informed consent prior to baseline examination. They were instructed to use either 0.05% bevacizumab or placebo eye drops four times daily, together with preservative-free artificial tears (0.18% sodium hyaluronate) at least four times daily, in both eyes, and to keep all medications at 4 °C. Participants in both groups were also advised to instill the investigational or placebo eye drops for 12 weeks. Sex, age, TBUT, Oxford scheme grade, Schirmer test, and ocular surface disease index (OSDI) score were obtained at baseline, after randomization. A well-trained research assistant determined the compliance of each subject. Emergency contact information was given to the participants, in case of any adverse effects during the trial.

Participants were randomized to receive either 0.05% bevacizumab eye drops or placebo by simple randomization with a 1:1 allocation ratio. The randomization sequence was put in an opaque, sealed envelope and kept by a research assistant. Packaging for the investigational eye drops and placebo was standardized and visually indistinguishable.

All investigators and participants were masked to treatment assignments for the duration of the participants’ involvement in the study. At the completion of the study, the research assistant unmasked the randomization sequence and forwarded the results to the study team for data analysis.

### Outcome measurement

The primary endpoint of this study was TBUT. The secondary endpoints were the proportion of responder in which the responder was defined as a clinically meaningful improvement (increase of 3 seconds or more) from baseline which was observed at week 12 in TBUT, OSDI score, Oxford scheme grade and Schirmer test. All outcome data were collected from the one eye (either left or right), that had the worse TBUT. Participants in both groups were examined at the beginning of the study and at follow-up visits at 1-, 4- and 12 weeks.

The OSDI score is obtained from a 12-item questionnaire designed to provide a rapid assessment of: symptoms of ocular irritation consistent with DED, and their impact on vision-related functioning. The research assistant asked each participant the OSDI questions at every visit [9]. TBUT was performed using the pre-cut 2% sodium fluorescein strip (Ophthalmic Technology Pvt. Ltd., India) which was wetted with one drop 0.9% normal saline; the excess saline was shaken off and then placed at the inferior fornix of the participant’s conjunctiva. Participants were instructed to blink naturally several times without squeezing. Within 10–30 seconds of the fluorescein strip instillation, the participants were asked for staring ahead without blinking. Afterwards, the researcher would observe the tear film over the entire cornea under slit-lamp with cobalt-blue light and recorded time in s between last complete blink and growing micelle. TBUT was evaluated three times per visit. The average TBUT was calculated. In addition, the Oxford scheme grade was obtained under cobalt-blue light after fluorescein staining. To grade the staining, the appearance of staining on the exposed interpalpebral region of conjunctivas and corneas was quantified using a chart comprising a series of panels. The severity designation from keratoconjunctival staining was rated mild (stage 0 or 1), moderate (stage 2 or 3), or severe (stage 4 or 5) [10]. Schirmer test was performed by placing Schirmer MARK BLU Tear Test^®^ strips (Optitech Eyecare, Allahabad, India) at the inferior fornix of the participant for 5 minutes, without anesthetic eye drops. After 5 minutes, the wetness of the filter paper was measured in millimeter (mm) from the initial fold. The sequence of outcome measurement which was the same throughout the study was OSDI question, TBUT, Oxford Scheme Grade, and Schirmer test, respectively. All efficacy outcome data were collected by one of the researchers (K.C.) at the same room which was assigned for conducting this study. The temperature of this room was set 25 °C throughout the study.

Participants were asked about adverse events at every visit, with the responses recorded by the research assistant.

### Sample size calculation

The necessary sample size was calculated from the data obtained from observation, prior to the study, of 14 DED patients (14 eyes) at an outpatient clinic. Mean ± standard deviation (SD) of TBUT among patients with DED who received conventional artificial tears was estimated to be 3.47 ± 1.75 s. A minimal clinically significant effect of adding bevacizumab 0.05% eye drops was defined by corneal specialists as a 3-s increase in TBUT. With a power of 90%, to be able to detect a difference of 3 s at a significance level of 0.05 (two-sided), 8 participants were needed in each group. To mitigate the risk of loss to follow-up, 2 participants were added per group (estimated drop-out rate of 20%). Adding the additional participants gave a recruitment target of at least 10 participants per group.

### Statistical analysis

To evaluate the difference between the two groups (bevacizumab and control groups) over time, we employed linear mixed-modeling with random intercept, with time as a categorical variable. Furthermore, to determine whether each outcome measure in the two groups changed differently over time, an interaction term between time and treatment group was included if the P-value for the interaction < 0.20 To control type-1 error, Scheffe’s method was used as a post hoc test for multiple comparisons. Fisher’s exact test was used to compare the proportion of responder between bevacizumab and control group.

All efficacy analyses were performed as intent-to-treat and involved all participants who were randomly assigned.

For all analyses, a level of 0.05 was adopted for statistical significance. Data were expressed as means with 95% confident intervals (CI). Statistical analyses were performed with Stata 15.1 (Stata Statistical Software: Release 15. College Station, TX: StataCorp LLC).

## Results

**Fig 1:**
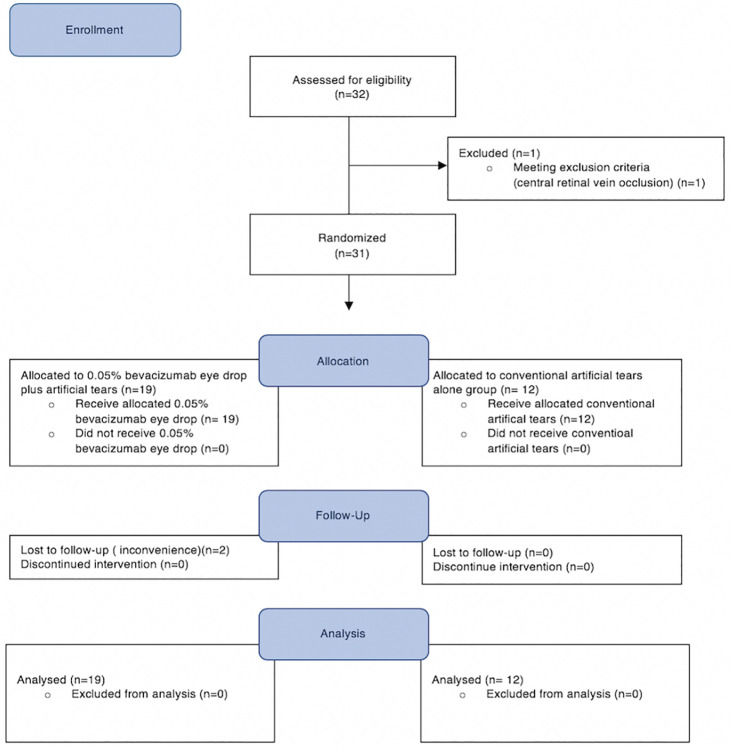
CONSORT 2010 flow diagram showing the flow of participants in the study entitled “Efficacy of topical bevacizumab 0.05% eye drops in dry eye disease”.

Thirty-two participants who were suffering from dry eye at a DEWS severity level 3 or 4 in at least one eye were recruited, but one participant was excluded due to another ocular condition (central retinal vein occlusion); that participant was then treated by a retina specialist. The remaining 31 participants were randomized into two groups (19 in the bevacizumab 0.05% eye drops group and 12 in the placebo group) between June, 2017 and November, 2017. Twenty-nine participants completed the 3-month study period but two participants attended only first visit because of the inconvenience of the multiple follow-ups (Fig 1). The baseline outcomes and characteristics for both groups are shown in Table 1.

**Table 1:** Baseline demographics and clinical characteristics.

	Bevacizumab 0.05% eye drops (n = 19)	Placebo (0.9% NSS) (n = 12)
Age in years, mean (SD)	52.63 (8.43)	53.50 (18.49)
Sex		
Male (%)	2 (10.53%)	2 (16.67%)
Female (%)	17 (89.47%)	10 (83.33%)
Postmenopausal female (%)	11 out of 17 (64.70%)	6 out of 10 (50%)
TBUT (s), mean (SD)	2.96 (1.11)	2.86 (0.96)
OSDI score, mean (SD)	30.04 (13.06)	20.41 (9.01)
Schirmer test (mm), mean (SD)	6.30 (5.56)	6.69 (8.15)
Oxford scheme grade (0–5), mean (SD)	1.16 (0.76)	1 (0.74)

NSS = normal saline solution; SD = standard deviation; Seconds = s; TBUT = tear break-up time; OSDI = Ocular Surface Index Score

### Efficacy

All efficacy outcome data are reported as predicted values from the final model of linear regression analysis, including an interaction term between time course and investigational product (Table 2). The interaction term was significant for TBUT, Oxford grading scheme and OSDI (P = 0.007, 0.16, and <0.001, respectively) and was added to the final model. The final model was reported without an interaction term (P = 0.47) for Schirmer test.

**Table 2:** Predicted mean values of all outcomes at 1-week, 4-week and 12-week visits.

	Baseline				1 week				4 weeks				12 weeks			
	Mean [95%CI]				Mean [95% CI]				Mean [95% CI]				Mean [95% CI]			
	Beva*		Placebo		Beva*		Placebo		Beva*		Placebo		Beva*		Placebo	
	Observed	Predicted	Observed	Predicted	Observed	Predicted	Observed	Predicted	Observed	Predicted	Observed	Predicted	Observed	Predicted	Observed	Predicted
TBUT (s)	2.96 [2.43,3.50]	2.96 [2.27,3.66]	2.86 [2.25,3.47]	2.86 [1.99,3.73]	5.06 [4.01,6.11]	5.10 [4.38,5.83]	3.61 [2.86,4.36]	3.61 [2.74,4.48]	5.20 [4.16,6.24]	5.24 [4.52,5.96]	3.00 [2.36,3.64]	3.00 [2.13,3.87]	5.12 [3.93,6.31]	5.16 [4.44,5.88]	2.97 [2.43,3.50]	2.97 [2.10,3.85]
Oxford Scheme Grade	1.16 [0.79,1.53]	1.16 [0.82,1.50]	1.00 [0.53,1.47]	1.00 [0.57,1.43]	0.47 [0.10,0.84]	0.49 [0.14,0.85]	0.83 [0.47,1.20]	0.83 [0.40,1.26]	0.59 [0.07,1.10]	0.61 [0.26,0.97]	0.67 [0.25,1.08]	0.67 [0.24,1.10]	0.65 [0.17,1.12]	0.67 [0.32,1.02]	0.50 [0.07,0.93]	0.50 [0.71,0.93]
OSDI score	30.04 [23.75,36.34]	30.04 [25.15, 34.94]	20.41 [14.69,26.14]	20.41 [14.25, 26.57]	16.74 [10.60,22.88]	16.49 [11.41, 21.58]	13.25 [9.77,16.73]	13.25 [7.09, 19.41]	15.48 [8.23,22.72]	15.23 [10.14, 20.32]	17.22 [11.32,23.12]	17.22 [11.06,23.38]	18.27 [12.12,24.41]	17.39 [12.21, 22.57]	17.38 [12.32,22.43]	16.67 [10.36,22.98]
Schirmer test (mm)	6.30 [2.58,7.94]	6.30 4.04,8.57]	8.33 [3.15,13.52]	6.69 [4.07,9.30]	7.20 [3.87,10.54]	6.89 [4.55,9.23]	6.83 [3.60,10.07]	7.27 [4.64,9.90]	6.83 [3.94,9.70]	6.97 [4.63,9.32]	7.58 [4.16,11.00]	7.35 [4.73,9.99]	6.69 [3.88,9.50]	5.39 [3.01,7.77]	4.33 [1.95,6.72]	5.77 [3.13,8.41]

CI = Confident interval; Beva* = Bevacizumab 0.05% eye drop group; TBUT = Tear break-up time; s = Second; OSDI = Ocular surface disease index

#### Tear Break-Up Time (TBUT)

**Fig 2:**
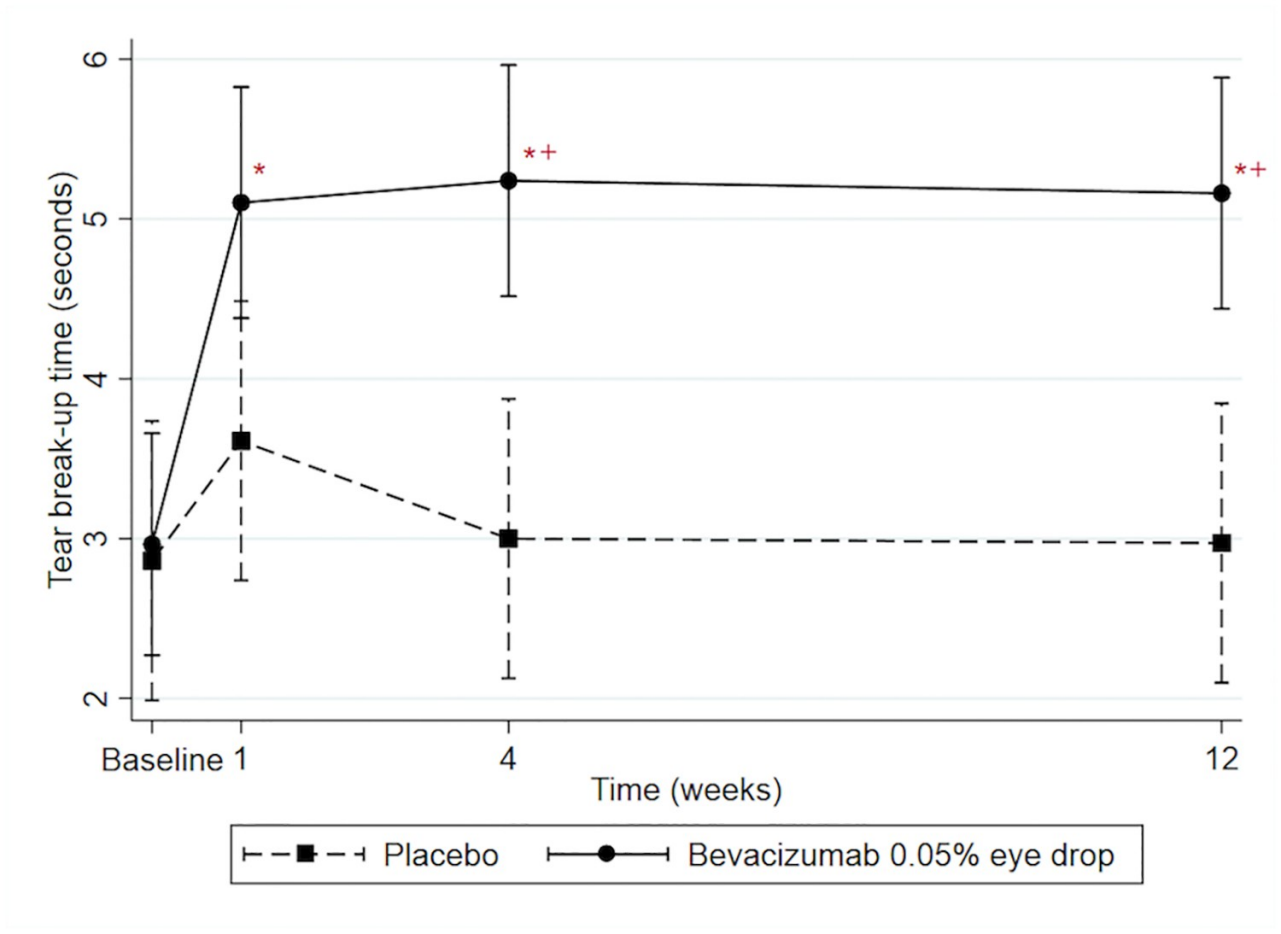
Predicted mean tear break-up time (TBUT) with 95% confidence intervals. * Represents significant difference compared with baseline within group (P<0.05). + Represents significant difference in the difference of TBUT between bevacizumab 0.05% eye drops group and placebo group compared with the difference between the two groups at baseline (P<0.05).

On average, TBUT in the bevacizumab group differed significantly from TBUT in the placebo group (1.51 s; CI = 0.60,2.41; P = 0.001). The differences in TBUT between two groups (bevacizumab vs placebo groups) at 4 weeks (2.24 s; CI = 0.07,4.41; P = 0.002) and 12 weeks (2.18 s; CI = 0.02,4.34; P = 0.003) were much longer than that at baseline (0.10 s; CI = -2.03,2.24) (Fig 2). However, the difference between two groups at 1 week (1.49 s; CI = -0.68,3.66; P = 0.11) did not significantly differ from that difference at baseline. Furthermore, TBUT at the 1-, 4- and 12-week visits significantly differed from baseline-TBUT within the bevacizumab group (2.14 s; CI = 1.13,3.14; P<0.001, 2.27 s; CI = 1.27,3.28; P<0.001, and 2.20 s; CI = 1.19,3.20; P<0.001, respectively). TBUT at the 1-, 4- and 12-week visits did not differ significantly from baseline-TBUT within the placebo group (0.75 s; CI = −0.46, 1.96; P = 0.39, 0.14 s; CI = −1.07, 1.35; P = 0.99, and 0.11 s; CI = −1.09, 1.32; P = 0.99, respectively).

#### Proportion of responders

A higher percentage of participants in the bevacizumab group (41.18%, 7 participants) experienced a clinically meaningful improvement in TBUT results (increase of 3 seconds or more) from baseline to week 12 as compared with the placebo group (0%, 0 participant). The difference of responder percentage between groups was statistically significant (P = 0.02).

#### Oxford grading scheme

**Fig 3:**
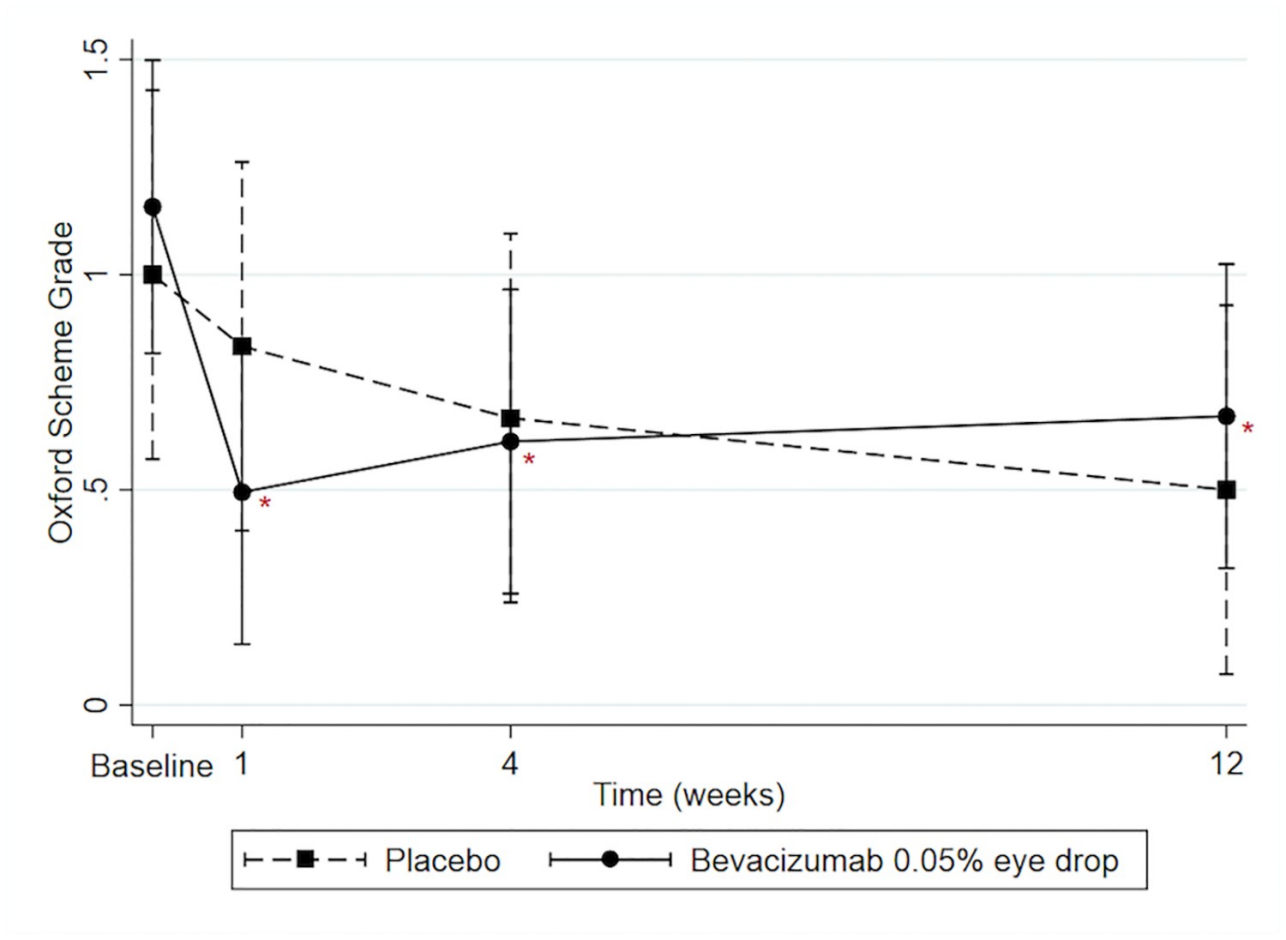
Predicted Oxford scheme grade (0–5) with 95% confidence intervals. * Represents significant difference compared with baseline within group (P<0.05).

On average, the Oxford scheme grade of the bevacizumab group did not differ significantly from the placebo group (-0.02; CI = -0.48,0.44; P = 0.95). The difference of Oxford scheme grade between two groups at the 1- (-0.34; CI = -1.40,0.72; P = 0.29), 4- (-0.05; CI = -1.12,1.01; P = 0.88) and 12-week (0.17; CI = -0.89,1.23; P = 1.00) follow-ups did not differ significantly from that difference at baseline (0.16; CI = -0.89,1.21) (Fig 3).

Within the bevacizumab group, the Oxford scheme grade at the 1-, 4- and 12-week visits differed significantly from that at baseline (−0.66; CI = −1.12,−0.21; P = 0.001, −0.55; CI = −1.00,−0.09; P = 0.01, and −0.49; CI = −0.94,−0.03; P = 0.03, respectively). Moreover, there was no significant difference of Oxford scheme grade between 1-week and 4-week (0.12; CI = -0.34,0.58; P = 0.92), 1-week and 12-week (0.18; CI = -0.29,0.64; P = 0.77), 4-week and 12-week (0.06; CI = -0.40,0.52; P = 0.99) in bevacizumab group. The Oxford scheme grades in the placebo group at the 1-, 4- and 12-week visits were not significantly less than the grade at baseline (−0.17; CI = −0.72,0.38; P = 0.87, −0.33; CI = −0.88,0.22; P = 0.41, and −0.50; CI = −1.05,0.05; P = 0.09, respectively).

#### Ocular Surface Disease Index (OSDI)

**Fig 4:**
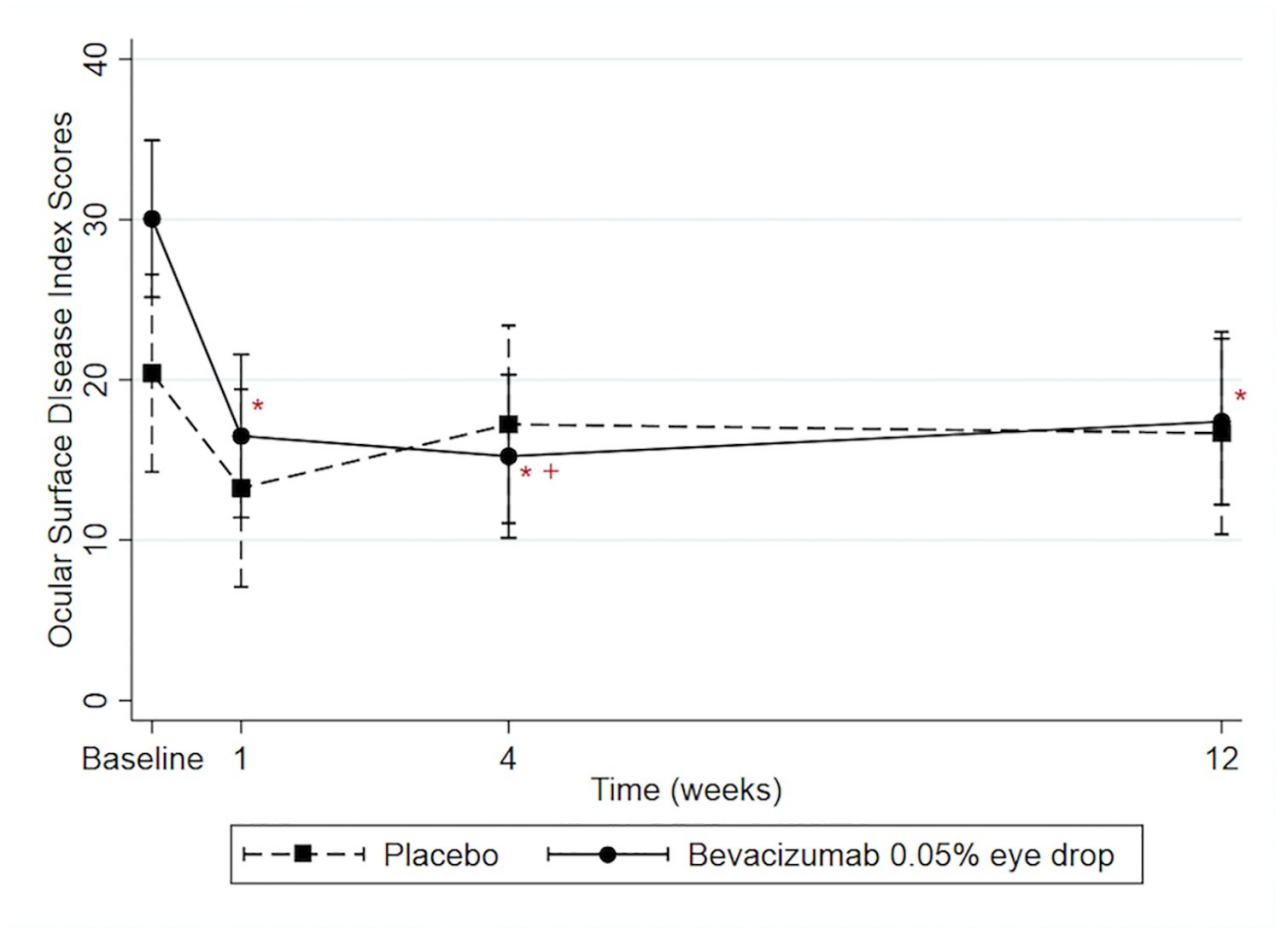
Predicted Ocular Surface Disease Index (OSDI) score (0–100) with 95% confidence intervals. * Represents significance difference compared with baseline within group (P<0.05). + Represents significance difference in the difference of OSDI scores between the bevacizumab 0.05% eye drops group and the placebo group compared with the difference between the two groups at baseline (P<0.05).

On average, the OSDI score of the bevacizumab group was not significantly less than the OSDI score of the placebo group (2.90; CI = −3.65,9.45, P = 0.39). The difference of OSDI score between two groups at the 4-week follow-up (−1.99; CI = −17.28,13.30; P = 0.02) significantly differed from that difference at baseline (9.63; CI = −5.43,24.69), but there was no significant difference at the 1- (3.25; CI = -12.04,18.54; P = 0.41) and 12-week follow-ups (0.72; CI = −14.90,16.34; P = 0.15) (Fig 4).

Within the bevacizumab group, the OSDI score at the 1-, 4- and 12-week follow-ups were significantly lower than score at baseline (−13.55; CI = −20.30,−6.80; P<0.001, −14.82; CI = −21.57,−8.07; P<0.001 and −12.65; CI = −19.54,−5.77; P<0.001, respectively). Conversely, the OSDI score in the placebo group at the 1-, 4- and 12-week follow-ups were not significantly less than the baseline score (−7.17; CI = −15.29,0.96; P = 0.11, −3.19; CI = −11.32,4.93; P = 0.75 and −3.74; CI = −12.10,4.61; P = 0.67, respectively).

#### Schirmer test

**Fig 5:**
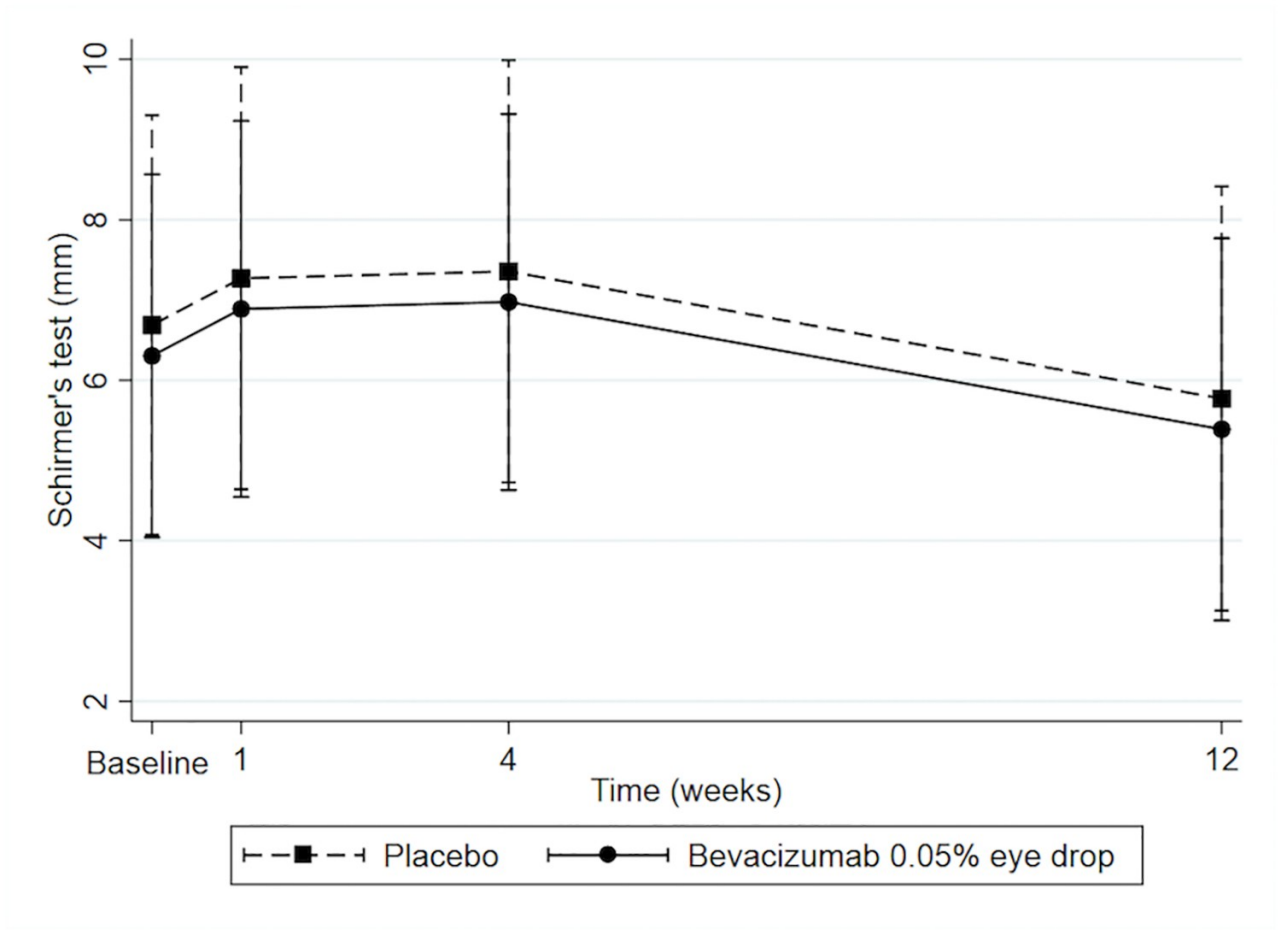
Predicted Schirmer test with 95% confidence intervals.

On average, the difference in Schirmer test values between the groups was insignificant (−0.38 mm; CI = −4.73,3.97; P = 0.10). The difference of Schirmer test between the bevacizumab and placebo groups at each visit did not significantly differ from that difference at baseline, as shown in Fig 5 (−0.38 mm; CI = −4.73,3.97, P = 0.10 for each visit). The Schirmer test at 1-, 4- and 12 weeks did not significantly differ from baseline within bevacizumab group (0.58 mm; CI = −2.94,4.11; P = 0.98, 0.67 mm; CI = −2.85,4.20; P = 0.96, and −0.92 mm; CI = −4.48,2.65; P = 0.92 respectively). Moreover, the Schirmer test at 1-, 4- and 12 weeks also did not significantly differ from baseline within placebo group (0.58 mm; CI = −2.94,4.11; P = 0.98, 0.67 mm; CI = −2.85,4.20; P = 0.96, and −0.92 mm; CI = −4.48,2.65; P = 0.92, respectively).

### Safety

Throughout the period of this study, no one contacted the investigators regarding an adverse event.

## Discussion

According to the TFOS DEWS II^™^, VEGF is a pro-inflammatory cytokine that can induce hemangiogenesis and lymphangiogenesis factors such as VEGF-A, VEGF-C and VEGF-D, as well as other inflammatory cytokines that are the core mechanisms of DED [7, 11, 12]. Lymphangiogenesis may serve as a channel for migration of corneal antigen-presenting cells to lymphoid tissues. As a result, autoreactive Helper T cell (T_H_)17 and T_H_1 cells, which are involved in DED immunopathogenesis, are generated in these lymphoid tissues [7]. Recently, VEGF has been studied, and anti-VEGF has been developed into various ophthalmic medications [2, 8]. Bevacizumab, a monoclonal antibody to VEGF-A, prevents attachment of VEGF-A to its receptors [13]. Previously, there was a study showing that systemic anti-VEGF-C treatment in mice could suppress inflammation and lymphangiogenesis in DED [7]. Since VEGF-A could recruit VEGF-C- and -D-producing macrophages, blockage of VEGF-A is believed to block lymphangiogenesis [11]. As a result, anti-VEGF-A treatment could inhibit angiogenesis and lymphangiogenesis, which are considered to be parts of the dry eye vicious cycle [1, 6, 13].

To our knowledge, there have been no studies about the effect of bevacizumab (anti-VEGF-A) in topical form on DED. Our study was the first randomized, controlled trial exploring the use of bevacizumab, in the form of 0.05% bevacizumab eye drops, for treating DED. Treatment with bevacizumab 0.05% eye drops in our study resulted in significant improvement in the primary outcome, TBUT, at 1-, 4- and 12 weeks. On the contrary, TBUT did not significantly improve in the placebo group. Furthermore, TBUT in the bevacizumab group was significantly longer than that in the placebo group at 4- and 12 weeks and the proportion of responder in bevacizumab group was significantly higher than the proportion of placebo group. To conclude, TBUT was significantly improved in the bevacizumab group. Some, but not all, of the secondary outcomes also improved. The Oxford scheme grade at all three follow-ups significantly decreased from baseline in the bevacizumab group, but there was no significant decrease in the placebo group. However, there was no statistically significant differences in Oxford scheme grade between the bevacizumab and placebo groups. Enriquez et al. illustrated the association between the inflammatory cytokines of DED, such as IL1-Ra, IL-6 and VEGF, and tear stability, tear production or ocular surface integrity [14]. Fraiselli et al. also reported that significantly decreased IL-1β, IL-6, and IL-8 levels in tears accompanied the improvement in corneal staining and TBUT after treatment with trehalose/hyaluronate tear substitute in dry eye patients [15]. The improvement in TBUT and Oxford scheme grade in the present study suggests that anti-VEGF treatment would decease inflammation and the cycle of DED, resulting in decreased corneal epitheliopathy and improved tear quality. As VEGF-A induces pro-inflammatory cytokines i.e. IL-1, IL-6 and TNF*α*, which impair the ocular surface epithelium, anti-VEGF-A treatment was believed to be able to suppress desiccating stress on the ocular surface epithelium, reduce inflammation, and finally lead to increased tear film stability and decreased corneal fluorescein uptake [7, 12].

OSDI scores significantly decreased at 1-, 4- and 12 weeks in the bevacizumab group, but not in the placebo group. However, OSDI scores in the bevacizumab group did not differ significantly from those in the placebo group. VEGF-A acted upon survival and neuronal axonal outgrowth of sensory neurons in a study of its role in chronic pain [16]. VEGF-A also played a role in inflammatory pain in early rodent studies [16] and was found in association with pathogenesis in neuropathic pain [17] and cancer pain [18]. According to a study by Belmonte et al., which was also published in the TFOS DEWS II^™^, pro-inflammatory cytokines may sensitize, directly induce ongoing nervous activity in, or increase activation of the corneal nerve terminal [19]. Enriquez et al. also reported an association between inflammatory cytokines and pain in dry eye patients [14]. The fact that VEGF induces pro-inflammatory cytokines may explain why bevacizumab 0.05% eye drops can improve OSDI scores in dry eye patients, i.e., by inhibiting VEGF-A, itself, and other pain-associated cytokines, including IL-1*β*, IL-17 and IL-18. [20] However, the complete details of the molecular inflammatory mechanisms are still unknown [19].

The improvements we observed in TBUT and OSDI results are in accordance with Jiang et al.’s study of subconjunctival bevacizumab injection in DED [2]. Improvement in corneal staining in the bevacizumab group in this study was not, however, in agreement with the Jiang et al. study, but was consistent with Goyal et al.’s study showing improvement in corneal staining after intraperitoneal injections of anti-VEGF antibody in mice [7].

In this study, there were improvements in TBUT, Oxford scheme grade and OSDI scores, but no improvement in Schirmer test which was found no significant change within either group within 12 weeks. Many reports have also shown an inconsistent association between signs and symptoms, and disagreement among signs in DED [21–24]. This study had a 12-week follow-up period, but it is possible that a longer period is needed to find enhancement of sensory-stimulated reflex tearing, leading to improved lacrimal gland function, like the cyclosporin-A mechanism, which might cause improvement in Schirmer test [25].

Most of parameters in this study showed improvement after bevacizumab 0.05% eye drop administration, especially TBUT, which is one of the important diagnostic signs in DED, according to the TFOS DEWS II^™^ [12]. While the Schirmer test results imply that the amount of tear film may not have improved, the tear quality was ameliorated, and so was the corneal coating. With the anti-inflammation property shown in our results, bevacizumab 0.05% eye drops may be considered as an alternative to steroids and cyclosporin-A, to decrease inflammation in DED, as steroids and cyclosporin-A can have adverse effects such as elevated intraocular pressure or cataract, and burning or stinging symptoms, respectively [25].

There was no any serious adverse events, in this 12-week study. The topical form may be a better choice than subconjunctival form for ease of use, less pain and fewer complications.

However, there were some limitations in our study. First, we were not able to control frequency (at least four times a day) of applying the artificial tears used by each participant, due to personal preferences, but we believe that their effects were minimal. Second, our study was a 12-week design, with 1-, 4- and 12-week visits. Even though this study showed positive results on multiple outcomes with bevacizumab 0.05% eye drops, the long-term effects need to be studied further. Finally, we believe that biological markers–IL-6 and VEGF–should be used to identify the biological effect of bevacizumab 0.05% eye drops in dry eye patients. A biological-markers study would support our clinical outcomes and the anti-inflammation efficacy of topical bevacizumab eye drops.

## Conclusion

Bevacizumab 0.05% eye drops produced statistically significant improvements in tear film stability, corneal staining and symptoms in dry eye patients. This is an innovative approach and should be considered as an alternative treatment for DED.

## Supporting information

S1 File CONSORT 2010 checklist of information to include when reporting a randomised trial*. (DOC)

S2 File (DOCX)

S1 Data (XLSX)
