# Supplementary material for: Efficacy of topical bevacizumab 0.05% eye drops in dry eye disease: A double-masked, randomized trial
Source: PLoS One. 2020 Jun 5;15(6):e0234186. doi: 10.1371/journal.pone.0234186 (PMC7274382; doi:10.1371/journal.pone.0234186)
Supplement: S2 File — (DOCX) [file pone.0234186.s002.docx]

**Research protocol**

Protocol title : **Efficacy of topical bevacizumab 0.05% eye drops in dry eye disease: A Double-Masked, Randomized Trial**

Investigator

Associate Professor Ngamjit Kasetsuwan, MD

Email : [ngamjitk@gmail.com](mailto:ngamjitk@gmail.com)

Assistant Professor Usanee Rienprayoon, MD

Email : [usaneer@gmail.com](mailto:usaneer@gmail.com)

Kanawat Chantaralawan, MD

Email: [kanawatc@gmail.com](mailto:kanawatc@gmail.com)

Lita Uthaithammarat, MD

Email: lita.t@chula.ac.th

Rational and review literature

As you walk into the ophthalmology clinic, both inpatient and outpatient, there are a great deal of patients with "dry eye" disease. The disease is associated with but not limited to corneal disease or from other ocular conditions. Moreover, it also includes patients with other medical conditions not directly related to ophthalmology such as Systemic Lupus Erythematosus (SLE) and Sjögren's syndrome (SS).

Although dry eye disease is a chronic condition with multiple causes in origin, it leads to multiple symptoms e.g. irritating sensation, blurry vision, etc. With the increase in tear osmolarity, it leads to inflammatory cytokine release - IL-1𝜶, IL-1β, TNF-𝜶, VEGF, EGD, and MMP-9. These inflammatory cytokines precede angiogenesis which trigger the vicious cycle of inflammation. Thus, anti-inflammatory or anti-angiogenesis medication is one of the keys to cut off this cycle.^1,2,3,5^

Current options of topical medication for dry eye disease:

1. Artificial tears

2. Gels and ointments

3. Anti-inflammatory agents

1) Topical Cyclosporine

2) Topical corticosteroids

3) Topical or systemic omega-3 fatty acid

4. Topical or systematic tetracyclines

5. Secretagogues - Diaquafosol

6. Autologous or umbilical cord serum

7. Systemic immunosuppressants

According to the study by Jiang X et el in September 2015, Subconjunctival Bevacicumab injection was found to statistically significant reduce dry eye symptoms and better features of tears properties as compared to the control group.^2^

Bevacicumab is an antibody against Anti-Vascular Endothelial Growth Factor receptor (Anti-VEGF receptor) which inhibits the function of VEGF compounds. They can be found in Vernal keratoconjunctivitis or dry eye disease patients.^3^ Furthermore, according to study by Kasetsuwan N et el in October 2015, they have demonstrated that Bevacicumab in topical form can suppress pterygium recurrence.^4^ From the mentioned-above reasons, we hypothesize that topical Bevacicumab can reduce symptoms and improve tears properties when compared to standard treatment in patients with dry eye disease.

**Related article and references**

1. 2007 Report of the International Dry Eye Workshop (DEWS)

2. Jiang X, Lv H, Qiu W, Liu Z, Li X, Wang W. Efficiency and safety of subconjunctival injection of anti-VEGF agent - bevacizumab - in treating dry eye. Drug Des Devel Ther. 2015;9:3043-50.

3. Abu El-Asrar A M, Al-Mansouri S, Tabbara K F, Missotten L, Geboes K, Immunopathogenesis of conjunctival remodelling in vernal keratoconjunctivitis. Eye (Lond), 2006;20:71–79.

4. Kasetsuwan N, Reinprayoon U, Satitpitakul V. Prevention of Recurrent Pterygium with Topical Bevacizumab 0.05% Eye Drops: A Randomized Controlled Trial. Clin Ther. 2015;37(10):2347-51.

5. Lopez-Miguel A, Teson M, Martin-Montanez V, Enriquez-de-Salamanca A, Stern ME, Gonzalez-Garcia MJ, et al. Clinical and Molecular Inflammatory Response in Sjogren Syndrome-Associated Dry Eye Patients under Desiccating Stress. Am J Ophthalmol. 2015.

**Objective**

To determine the efficacy of Bevacizumab eye drop in patients diagnosed with dry eye comparing with placebo group.

**Method**

**Study design**

Randomized, double masked, controlled trial

**Participants**

Participants were recruited from the outpatient clinic of the Department of Ophthalmology, King Chulalongkorn Memorial Hospital, and evaluated for the eligibility criteria.

Inclusion criteria

Age between 18 and 80 years

Tear break-up time (TBUT) ≤ 5 seconds

Ability and willingness to comply with the treatment/follow-up schedule and requirements

Ability to provide informed consent.

Exclusion criteria

Presenting with mild or moderate DED condition (severity level 1 or 2 according to DEWS 2007 report)^1^

Having history or presence of non-DED ocular surface disorder or structural abnormalities involving tear secretion or evaporation i.e. trichiasis or entropion

Having any other current active eye disease other than DED that required the use of ophthalmic medication

Presence with pterygium or pinguecula; any inflammation in the iris or anterior chamber

Glaucoma

Systemic conditions that affect the health of the ocular surface

History of bevacizumab contraindication

Using any topical medication other than artificial tears within the past 3 months

Using drugs that may interfere with tears production, i.e. anti-depressive, anticholinergics, anti-histamine medication, antihypertensive, calcium channel blocker, antacids, systemic corticosteroids, and retinoids within the past 3 months

Previous ocular surgery or contact lens use within the past 6 months.

Female of child bearing potential were excluded from participation in the study if they were pregnant.

**Sample Size Calculation**

The necessary sample size was calculated from the data obtained from observation, prior to the study, of 14 DED patients (14 eyes) at an outpatient clinic. Mean ± standard deviation (SD) of TBUT among patients with DED who received conventional artificial tears was estimated to be 3.47 ± 1.75 s. A minimal clinically significant effect of adding bevacizumab 0.05% eye drops was defined by corneal specialists as a 3-s increase in TBUT. With a power of 90%, to be able to detect a difference of 3 s at a significance level of 0.05 (two-sided), 8 participants were needed in each group. To mitigate the risk of loss to follow-up, 2 participants were added per group (estimated drop-out rate of 20%). Adding the additional participants gave a recruitment target of at least 10 participants per group.

Observational TBUT at out patient clinic of Department of Ophthalmology

| **ID** | **Laterality** | **TBUT** | | | | | **Average** |
| --- | --- | --- | --- | --- | --- | --- | --- |
| 1 | RE |  |  | | | 4.53 | 4.53 |
|  | LE |  |  | | | 2.22 | 2.22 |
| 2 | RE | 6 | 4 | | | 5 | 5 |
|  | LE | 3 | 3 | | | 5 | 3.666667 |
| 3 | RE | 1 | 1 | | | 1 | 1 |
|  | LE | 1 | 1 | | | 2 | 1.333333 |
| 4 | RE | 6 | | 6 | 5 | | 5.666667 |
|  | LE | 3 | | 4 | 3 | | 3.333333 |
| 5 | RE | 1 | | 1 | 2 | | 1.333333 |
|  | LE | 3 | | 3 | 2 | | 2.666667 |
| 6 | RE | 2 | | 2 | 3 | | 2.333333 |
|  | LE | 2 | | 2 | 4 | | 2.666667 |
| 7 | RE | 3 | | 3 | 3 | | 3 |
|  | LE | 4 | | 5 | 6 | | 5 |
| 8 | RE | 4 | | 4 | 4 | | 4 |
|  | LE | 4 | | 4 | 4 | | 4 |
| 9 | RE | 3 | | 3 | 2 | | 2.666667 |
|  | LE | 2 | | 3 | 2 | | 2.333333 |
| 10 | RE | 6 | | 5 | 7 | | 6 |
|  | LE | 5 | | 8 | 5 | | 6 |
| 11 | RE | 2 | | 2 | 3 | | 2.333333 |
|  | LE | 2 | | 2 | 2 | | 2 |
| 12 | RE | 1 | | 2 | 1 | | 1.333333 |
|  | LE | 1 | | 1 | 1 | | 1 |
| 13 | RE | 6 | | 4 | 5 | | 5 |
|  | LE | 3 | | 3 | 5 | | 3.666667 |
| 14 | RE | 2 | | 2 | 3 | | 2.333333 |
|  | LE | 3 | | 3 | 3 | | 3 |

**Methodology**

1. All information about all detail of the study will be informed to the participants and inform consent will be collected.
2. Demographic data and baseline characteristic of all participants will be recorded at the beginning day of the study including

- Age
- Sex
- Underlying diseases, past surgical history, current medication
- Baseline ocular characteristics: Tear break-up time (TBUT), Ocular Surface Disease Index (OSDI) score, Oxford Scheme Grade (staining), and Schirmer test with topical anesthetic eye drop (Schirmer 1 test)

1. All participants will be randomized to receive either bevacizumab eye drop or placebo eye drop by simple randomization.
2. All randomization code will be kept at research assistants in opaque sealed envelop.
3. The bevacizumab eye drops, supplied as a sterile, preservative-free, clear aqueous solution containing 0.05% bevacizumab, will be prepared from standard bevacizumab solution (Avastin, Genentech Inc, South San Francisco, CA) for intravenous infusion, diluted in 0.9% normal saline solution at the hospital’s Pharmacy Department. Placebo is prepared from 0.9% normal saline solution alone.
4. After all baseline data are collected, research assistant (the other one) will inform to all participants to use eye drop four time daily. Moreover, preservative-free artificial tears (0.18% sodium hyaluronate) will be prescribed to all participants and they have to use it at least four times a day. All eye drops will be informed to be kept at 4 degree celcius in refrigerator and all participant have to get new eye drop every two weeks.
5. On every follow-up visit, one investigator will exam TBUT, OSDI score, Oxford Scheme Grade, and Schirmer test of all participant. Research assistant will determine compliance of using eye drop, and record the frequency of artificial tears use and side-effect event after using eye drop.

**Follow-up**

1. Pre topical Bevacizumab

2. 1 week after topical Bevacizumab regimen starts

3. 1 month after topical Bevacizumab regimen starts

4. 3 months after topical Bavacizumab regimen starts

**Outcome**

**Primary outcome**

- Slit-lamp examination Tear break-up Time (TBUT)

**Secondary outcome**

- Proportion of responder in which the responder was defined as a clinically meaningful improvement (increase of 3 seconds or more) from baseline which was observed at week 12 in TBUT,
- Ocular Surface Disease Index (OSDI) score
- Oxford scheme grade
- Schirmer test (mm)

**Statistical analysis**

To evaluate the difference between the two groups (bevacizumab and control groups) over time, we will employ linear mixed-modeling with random intercept, with time as a categorical variable. Furthermore, to determine whether each outcome measure in the two groups changed differently over time, an interaction term between time and treatment group will be included if the P-value for the interaction < 0.20 To control type-1 error, Scheffe’s method will be used as a post hoc test for multiple comparisons. Fisher’s exact test will be used to compare the proportion of responder between bevacizumab and control group.

**Ethical consideration**

Respect of person

Beneficence/non-maleficence

Justice

**Risks of this project**

- Compliance of patients due to socioeconomic problems or time constraint

**Benefits from this project**

- To know the safety and efficacy of topical Bevacicumab in patients with dry eye disease

**Cost structure**

| **Cost structure** | **Amount (Baht)** |
| --- | --- |
| Pharmacologist | 2,000 |
| Data management personnel | 2,000 |
| Statistician | 2,000 |
| Translator/Editor | 2,000 |
| Miscellaneous | 5,000 |
| Lissamine green, fluorescein stain | 30,000 |
| Bevacicumab eye drop | 350,000 |
| Total | 393,000 |
